# Porcine liver decomposition product-derived lysophospholipids promote microglial activation *in vitro*

**DOI:** 10.1038/s41598-020-60781-1

**Published:** 2020-02-28

**Authors:** Tamotsu Tsukahara, Hisao Haniu, Takeshi Uemura, Yoshikazu Matsuda

**Affiliations:** 1 Department of Pharmacology and Therapeutic Innovation, Nagasaki University Graduate School of Biomedical Sciences, 1-14 Bunkyo-machi, Nagasaki, 852-8521 Japan; 2 Institute for Biomedical Sciences, Shinshu University Interdisciplinary Cluster for Cutting Edge Research 3-1-1 Asahi, Matsumoto, Nagano 390-8621 Japan; 3 Division of Gene Research, Research Center for Supports to Advanced Science, Shinshu University 3-1-1 Asahi, Matsumoto, Nagano 390-8621 Japan; 4 Division of Clinical Pharmacology and Pharmaceutics, Nihon Pharmaceutical University, Ina-machi, Saitama 362-0806 Japan

**Keywords:** Phospholipids, Cellular neuroscience

## Abstract

Cognitive impairments such as dementia are common in later life, and have been suggested to occur via a range of mechanisms, including oxidative stress, age-related changes to cellular metabolism, and a loss of phospholipids (PLs) from neuronal membranes. PLs are a class of amphipathic lipids that form plasma membrane lipid bilayers, and that occur at high concentrations in neuronal membranes. Our previous study suggested that a porcine liver decomposition product (PLDP) produced via protease treatment may improve cognitive function at older ages, by acting as a rich source of PLs and lysophospholipids (LPLs); however, its specific composition remains unclear. Thus, the present study used a novel liquid chromatography electrospray ionization tandem mass spectrometric (LC-MS/MS) protocol to identify the major PLs and LPLs in PLDP. Furthermore, it assessed the effect of identified LPLs on microglial activation *in vitro*, including cell shape, proliferation, and cell morphology. The results of the conducted analyses showed that PLDP and PLDP-derived LPLs concentration-dependently modulate microglial activation *in vitro*. In particular, lysophosphatidylcholine (LPC) concentration-dependently promotes cell morphology, likely via effects mediated by the enzyme autotaxin (ATX), since inhibiting ATX also promoted cell morphology, while conversely, increasing ATX production (via treatment with high levels of LPC) abolished this effect. These findings suggest that LPC is likely neuroprotective, and thus, support the importance of further research to assess its use as a therapeutic target to treat age-related cognitive impairments, including dementia.

## Introduction

Rapid population aging and age-related disease represent an increasing global health-care burden. Dementias are the most common cause of significant late-onset cognitive decline, and affect approximately 50 million people worldwide, with nearly 10 million new cases diagnosed annually^1^. There are many different forms of dementia; for example, Alzheimer’s disease is the most common form, and contributes to 60–70% of cases. Other major forms include vascular and Lewy body dementia, and a group of diseases that together comprise frontotemporal dementia^2^. Patients with mild cognitive impairment (MCI) are at risk of developing dementia; however, in some individuals, MCI reverts to normal cognition or remains stable. While clinical studies are currently being conducted to identify novel treatments to improve symptoms and/or prevent or delay the progression of MCI to dementia, no drugs or other treatments have yet been specifically approved for MCI. Lipids are increasingly recognized for their roles in neuronal function in the brain^3,4^; for example, the lipid fraction of mammalian membranes consists of glycerophospholipids, sphingolipids, and cholesterol^4^. We previously reported that a porcine liver decomposition product (PLDP) induces a significant increase in the Hasegawa’s Dementia Scale-Revised (HDS-R) score in a randomized, double-blind, placebo-controlled study in healthy humans^5^. Our previous work documented that PLDP produced via protease treatment is a good source of a class of lipids called phospholipids (PLs) that are crucial for normal cell membrane and neurotransmitter function, and thus, can enhance cognitive function at older ages^5,6^. The exact mechanism by which PLDP improves Delayed Recall, Visual Recall I, and Visual Recall II is unclear. One possibility is that neuro-functional PLs, which are contained in PLDP, may play an important role in improving brain function. PLDP mainly includes PLs, such as PC, PE, PI, PS, PA, SM and LPC. Reduced PL levels have been previously shown to be caused by a loss of PL precursors or choline, or by increased PL metabolism^7^. Choline is essential for cognitive function, because it is both the precursor for acetylcholine, and required for intracellular phosphatidylcholine (PC) synthesis. Accordingly, choline supplementation has been shown to exert profound effects on brain development, function, and aging^8–11^, and dietary PC supplements have been shown to enhance memory and learning capacity in middle-aged and older men^12^. However, choline transport into the brain is not very efficient, and tends to decline with age^13–15^. Previous attempts to treat dementia and cognitive impairment with choline supplements such as PC have had little success. For example, a randomized trial that compared PC with a placebo treatment did not find that the former had any significant effect^16^, while another study that assessed the effect of administering phosphatidylserine (PS) supplements to elderly patients 12 weeks did not reveal any resulting significant cognitive improvement^17^. Various isoforms of PLA_2_ enzyme have been reported to hydrolyze PC, phosphatidylserine (PS), and phosphatidylethanolamine (PE) at the sn-2 position to form lysophospholipids (LPLs), including lyso-PC (LPC), lyso-PS (LPS), and lyso-PE (LPE), respectively^18^. LPLs mediate signaling, proliferation, neural activity, and inflammation to mediate a variety of important processes, including the pathogenesis of cerebral ischemia, vascular dementia, and Alzheimer’s disease^19–23^. They are small bioactive lipid molecules that include only a single fat-chain on the 1-carbon, and are structurally classed as either lysophosphatidic acid (LPA), lysophosphatidylcholine (LPC), lysosphingomyelin (SPC), or sphingosine 1-phosphate (S1P)^24^. LPLs such as LPA and LPC are important bioactive lipids in the brain^25,26^; for example, LPA is derived from membrane PLs, and is the most biologically active LPL. In all the other LPLs, the phosphate group is esterified to an alcohol or amino-alcohol group to produce the various LPL species, e.g. LPC (which is the most naturally abundant LPL), or LPE. A previous study conducted using an *in vitro* model of excitotoxicity using glutamate treatment reported that both LPC and lysophosphatidylinositol (LPI) were protective when administered prior to, but not when they were applied only during or after glutamate exposure^19^. In fact, LPLs are now recognized as essential bioactive lipids that are involved in a large variety of both normal and pathological processes, such as neurogenesis, vascular development, and the regulation of metabolic disease^19^. A previous study by our research group supports the importance of further research into the potential use of PL supplementation (in PLDP) to prevent chronic and age-related disease^5^; thus, the present study used a novel liquid chromatography electrospray ionization tandem mass spectrometric (LC-MS/MS) protocol to identify major LPLs in PLDP, and furthermore assessed the effects of identified PLDP-derived LPLs on microglial activation *in vitro*.

## Materials and Methods

### Materials

PLDP was obtained from YAEGAKI Bio-industry, Inc. (Himeji, Japan). The extracted PLDP components have been previously reported^5^. Charcoal-Dextran stripping of PLDP prepared as follows. Activated charcoal (Fisher Chemicals, D127–500, 10 g/L) and Dextran (Sigma, D8821, 1 g/L) was added to PLDP (15 mL) followed by mixing at 56 °C for 45 min and centrifugation at 4000 rpm for 20 min and the supernatants were test for cell morphological change. A PL kit including LPC, LPE, phosphatidylinositol (PI), sphingomyelin (SM), PS, LPS, PC, PE, cardiolipin (CL), phosphatidic acid (PA), LPS, and CB was purchased from Olbracht Serdary Research Laboratories (Toronto, Canada). Murine microglial BV-2 and SIM-A9 cells were provided from Dr. Hiroshi Ueda (Kyoto University). Fetal bovine serum, poly-D-lysine, penicillin-streptomycin, and other chemicals were purchased from Sigma (St. Louis, MO, USA). Anti-ATX (GTX106209) antibody was purchased from GeneTex Inc. (Irvine, CA, USA). Anti-β-actin (sc-47778) antibodies were purchased from Santa Cruz Biotechnology (Santa Cruz, CA, USA). 18:1 lyso NBD-PC was purchased from Avanti Polar Lipids (Alabaster, AL, USA). The ATX inhibitor BI-2545 was provided by Boehringer Ingelheim (opnMe).

### Cell culture maintenance and treatment

BV2 and SIM-A9 cells were cultured (37 °C, 5% CO_2_) in Dulbecco’s modified Eagle’s medium (DMEM; Nacalai, Kyoto, Japan) that was supplemented with 10% fetal bovine serum (FBS), and used in experiments between passages 3–6^27^.

### Preparation of PLs from PLDP

Total lipids were extracted from PLDP using the Bligh and Dyer method^28^. An acidic HCl solution was used to improve acidic PL recovery, as per the protocol established by Honeyman^29^ (modified from Lloyd’s method^30^). In brief, 100 μL of PLDP was mixed with 0.375 ml of chloroform/methanol/12 N HCl (2/4/0.1 v/v). After thorough mixing, 0.125 ml of chloroform was added to this mixture, and the resulting solution was vortexed for 30 s. Next, 0.125 ml of water was added, and the solution was again vortexed for 30 s, before being centrifuged (1,000 rpm, 10 min). The lower chloroform layer was then removed and transferred to a glass tube for evaporation. A comprehensive PL analysis was conducted as described previously^31,32^. After final centrifugation, an aliquot of the lower organic phase was evaporated in N_2_, and the resulting residue then dissolved in methanol. To measure PA, PS, and PI levels, another aliquot of the same lipid extract was added with an equal volume of methanol, before being loaded onto a DEAE (diethylaminoethyl cellulose) column (Santa Cruz Biotechnology) that was pre-equilibrated with chloroform. After successive washes with chloroform/methanol (1:1 v/v), the acidic PLs were eluted with chloroform/methanol/HCl/water (12:12:1:1 v/v), evaporated, and the resulting residue was resolved in methanol, and subjected to a methylation reaction with TMS-diazomethane prior to LC-MS/MS analysis^33^.

### PL assay

PL levels were assessed using the molybdenum blue method^34^. In brief, extracted whole PLs were transferred into clean glass tubes, and the solvent was completely evaporated. Next, 0.65 ml of perchloric acid was added to each, before the tubes were heated (100 °C) for approximately 30 min (until the yellow color disappeared). Once cool, 3.3 ml water, 0.5 ml of molybdate solution, and 0.5 ml of ascorbic acid solution were added in order, and the solution was vortexed between each addition. The tubes were then placed into a boiling water bath for 5 min, before the absorbance (at 800 nm) of the cooled samples (including standards) was measured.

### MS analysis

As LC-MS/MS analysis was performed using the UltiMate 3000 LC system (Thermo-Fisher Scientific) and an HTC PAL autosampler (CTC Analytics)^32^. Briefly, 10 μL aliquots of lipid samples were injected and separated on Waters X-Bridge C18 columns (3.5 mm, 150 mm × 1.0 mm i.d.) at room temperature (25 °C) using a gradient solvent system comprising: mobile phase A (isopropanol/methanol/water (5/1/4 v/v/v) supplemented with 5 mM ammonium formate and 0.05% ammonium hydroxide)/mobile phase B (isopropanol supplemented with 5 mM ammonium formate and 0.05% ammonium hydroxide) ratios of 70%/30% (0 min), 50%/50% (0–2 min), 20%/80% (2–13 min), 5%/95% (13–30 min), 95%/5% (30–35 min) and 70%/30% (35–45 min)^35^. The flow rate utilized was 20 μL/min. PL species were measured via selected reaction monitoring (SRM; positive ion mode) with a triple-stage quadrupole mass spectrometer (TSQ Vantage AM, Thermo-Fisher Scientific). The characteristic fragments of individual PLs were detected via a product ion scan (MS/MS mode). Chromatographic peak areas were used to perform a comparative quantitation of the various molecular species (e.g. 38:6, 40:6) within each PL class (e.g. PA, PC)^36^.

### Measurement of cell proliferation and morphological change

Cells were seeded (5 × 10^3^ cells/well) in 96-well plates, and PLDP (diluted to 0.0625, 0.125, 0.25, 0.5, or 1%) or PL standards were added to well. After 24 h incubation (37 °C, 5% CO_2_), cell proliferation was determined using a Cell Counting Kit-8 (Dojindo, Kumamoto, Japan). After a further 24 h incubation, 10 µL of the Cell Counting Kit-8 solution was added to each well. The plates were then incubated for 2 h, before orange formazan dye levels were determined by measuring absorbance at 450 nm using a microplate reader (Awareness Technology, Westport, CT, USA). Assessment of morphological change in microglial cells were contiguous phase-contrast digital images of the entire well at 20× and 40× objective were acquired using a Olympus CKX41 microscope mounted with digital imaging camera (Nikon, Japan). The morphological change of randomly selected microglia in each well were then assessed by an independent observer using computer-assisted imaging^37^.

### Quantitative real-time reverse transcription polymerase chain reaction (RT-PCR)

RT-PCR analysis was performed as we previously described^38^. BV-2 cells were seeded (1 × 10^5^ cells/well) in 12-well plates, and PLDP (diluted to 0.0625, 0.125, 0.25, 0.5, or 1%) or PL standards were added to each well. After 24 h, the total RNA was extracted from cells using TRIzol (Invitrogen), and a NucleoSpin RNA II Kit (Takara, Otsu, Japan). Next, 0.5 µg of total RNA was converted to cDNA using a ReverTra Ace qPCR RT Kit (Toyobo) as per the manufacturer’s instructions. mRNA levels were quantified using the QuantStudio 12 K Flex Real-time PCR system (ThermoFisher Scientific). All PCRs (volume, 10 μL) were performed in 384-well PCR plates (FrameStar, 4titude), using GeneAce SYBR qPCR MIX α with low ROX (Nippon Gene, Toyama, Japan), and the specified primers (Table 1). Utilized cycling conditions were as follows: 95 °C for 10 min (polymerase activation), followed by 40 cycles of 95 °C for 15 s, 55 °C for 15 s, and 72 °C for 30 s. After amplification, the samples were slowly heated from 55 °C to 95 °C, and fluorescence was measured continuously to obtain a melting curve. Relative mRNA levels were quantified using the formula 2^−ΔΔCq^, where ΔCq is the difference between the threshold cycle of a target cDNA and an endogenous reference cDNA.

**Table 1:** List of forward primers and reverse primers used for amplification using real-time PCR.

Gene Name	Primer (Forward)	Primer (Reverse)
β-actin	AGGCACCAGGGCGTGAT	GCCCACATAGGAATCCTTCTGAC
IL-1β	GGCTGGACTGTTTCTAATGC	AGCTTCTCCACAGCCACAAT
IL-4	ACTTGAGAGAGATCATCGGCA	AGCTCCATGAGAACACTAGAGTT
IL-6	CTGCAAGAGACTTCCATCCAG	AGTGGTATAGACAGGTCTGTTGG
IL-10	AAGGCAGTGGAGCAGGTGAA	CCAGCAGACTCAATACACAC
IL-12	GTCCTCAGAAGCTAACCATCTC	CCAGAGCCTATGACTCCATGTC
TGF-β	CCCAGCATCTGCAAAGCTC	GTCAATGTACAGCTGCCGCA
TNF-α	ACGGCATGGATCTCAAAGAC	GTGGGTGAGGAGCACGTAGT

### BNPP assay

The ability of ATX to cleave the nucleotide-like substrate BNPP was determined by measuring the generation of the yellow product, p-nitrophenyl. human ATX was incubated with 10 μM LPA, LPC or vehicle control) in assay buffer [50 mM Tris (pH 8.0), 140 mM NaCl, 1 mM MgCl_2_, 1 mM CaCl_2_, and 5 mM KCl] for 15 minutes at 37 °C before the addition of BNPP to 1.5 mM. After an additional 1 h incubation at 37 °C, the liberated p-nitrophenyl was detected using a microplate reader (Awareness Technology, Westport, CT, USA) by measuring the absorbance at 405 nm.

### Western blot

Western blot analysis was performed as we previously described^27^. Cells were lysed with RIPA buffer (ATTO, Tokyo, Japan), and protein concentration was determined using the Bradford method (Dojindo, Japan). Proteins were separated on 4–20% sodium dodecyl sulfate-polyacrylamide gel electrophoresis gels and electro-transferred to polyvinylidene fluoride membranes (BIO-RAD, CA, USA). The membranes were blocked in Block Ace (DS Pharma Biomedical Co. Ltd., Osaka, Japan) for 1 h and were then incubated with primary antibodies in Tris-buffered saline-Tween 20 with 5% Block Ace for 12 h at 4 °C. Unbound primary antibodies were washed off, and the membranes were then incubated with horseradish peroxidase-conjugated secondary antibodies. Bands were visualized with EzWestLumi plus (ATTO) and images were obtained using a ChemiDoc Touch Imaging System (Bio-Rad, Hercules, CA, USA).

### Statistical analysis

Data were analyzed using an unpaired Student’s *t*-test or one-way ANOVA, followed by Newman-Keuls post hoc testing, using GraphPad Prism Ver. 5.01 (GraphPad Software Inc.). Results were expressed as the mean ± SEM. A p-value <0.05 was considered to indicate statistical significance^27^.

## Results

### PLDP promotes microglial growth and morphological change *in vitro*

**Figure 1:**
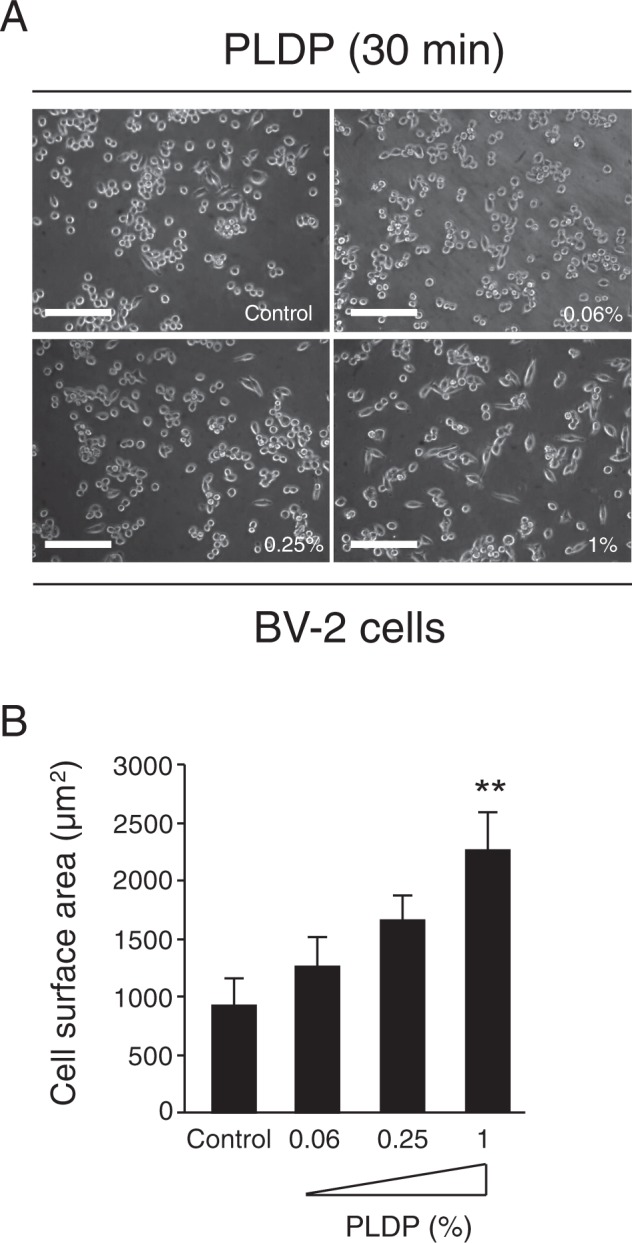
Modulation of neuronal microglial cell size by porcine liver decomposition product (PLDP) treatment. (**A**) BV-2 cells (≥3 fields/sample) were analyzed and counted as described. (**B**) Quantification of cell surface after treatment with the indicated concentrations of porcine liver decomposition product (PLDP). Cell surface area was quantified by ImageJ software. Data are expressed as mean ± S.E. n = 3, **P < 0.01. Bars, 100 μm.

**Figure 2:**
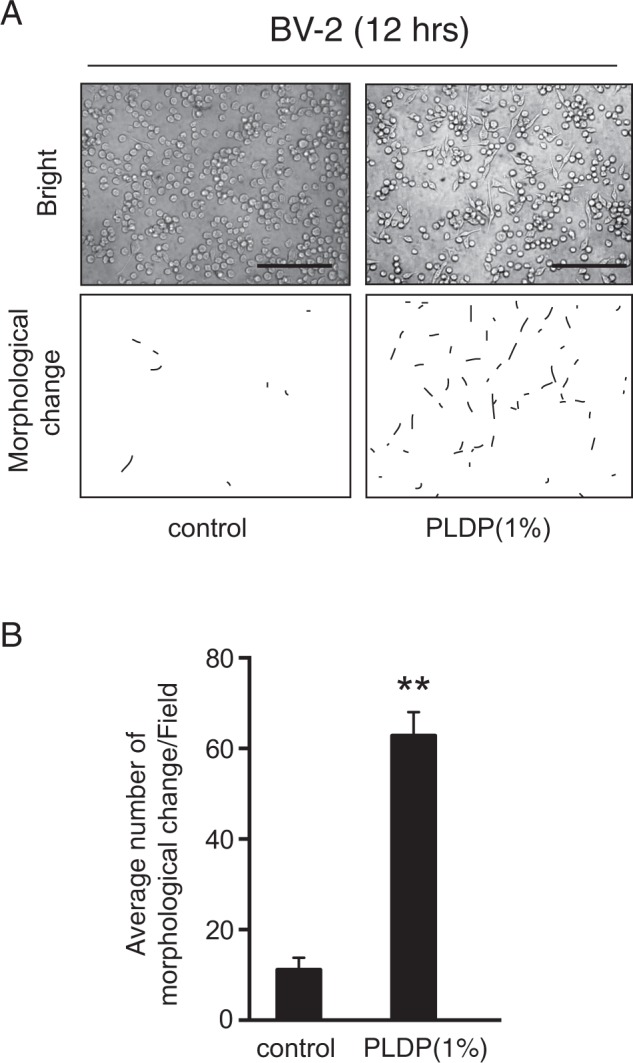
Porcine liver decomposition product (PLDP) induced morphological change (*microglial processes*) in microglia. (**A**) Representative images of BV-2 microglial cells treated with 1% PLDP for 12 h. Bright field (upper figures) and representative drawings of the morphological change (lower figures) are shown. Bars, 100 μm (**B**) The number of cells exhibiting morphological change was measured after 1% PLDP for 12 h, before morphological change (in ≥ 3 fields/sample) was analyzed as described.

To validate our previous findings, we first assessed whether and how PLDP administration affects microglial cell size (i.e. cell mass), proliferation, and cell morphology (i.e. microglial processes). The results of the conducted analyses showed that PLDP (0.06, 0.25, and 1%) treatment increased BV-2 cell size within 30 min (Fig. 1A,B). Moreover, microglial processes was induced at a markedly faster rate in PLDP-treated than control murine BV2 cells (Fig. 2A,B), suggesting that PLDP treatment enhanced cell morphology.

### PLDP composition

**Figure 3:**
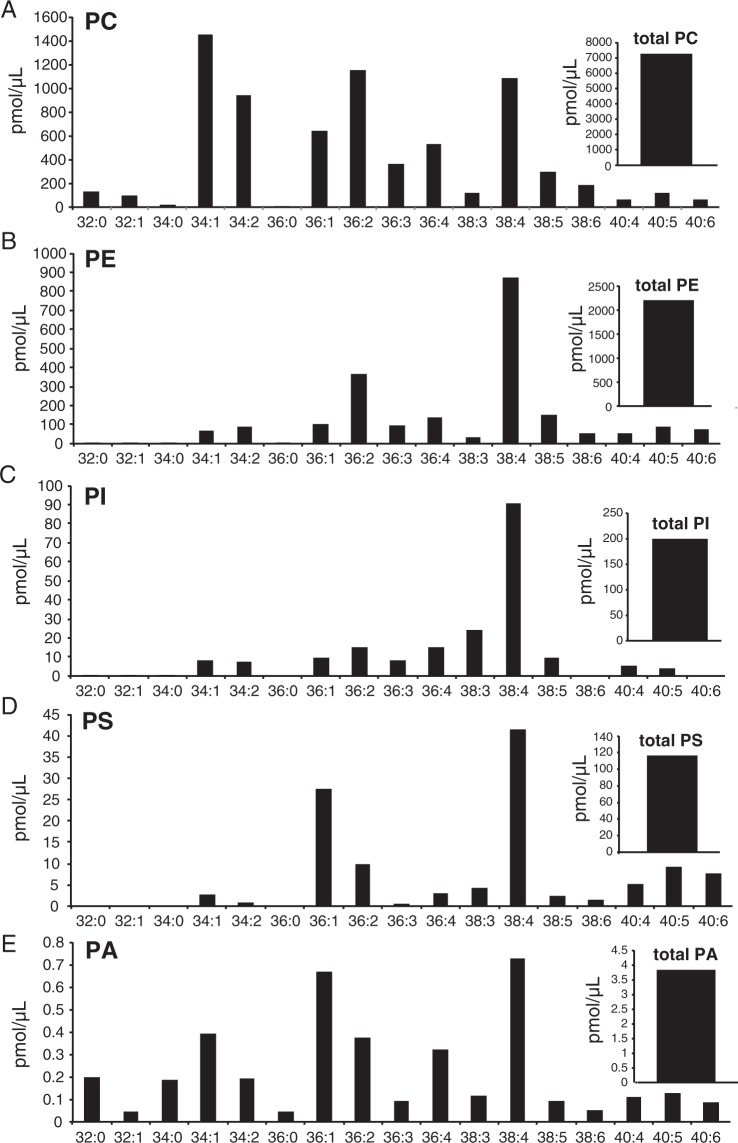
Liquid chromatography electrospray ionization tandem mass spectrometric (LC-MS/MS) analysis of porcine liver decomposition product (PLDP) phospholipid (PL) composition. PL species in PLDP were identified (via selected reaction monitoring), including (**A**) phosphatidylcholine (PC), (**B**) phosphatidylethanolamine, (**C**) phosphatidylinositol (PI), (**D**) phosphatidylserine (PS), and (**E**) phosphatidic acid (PA).

**Figure 4:**
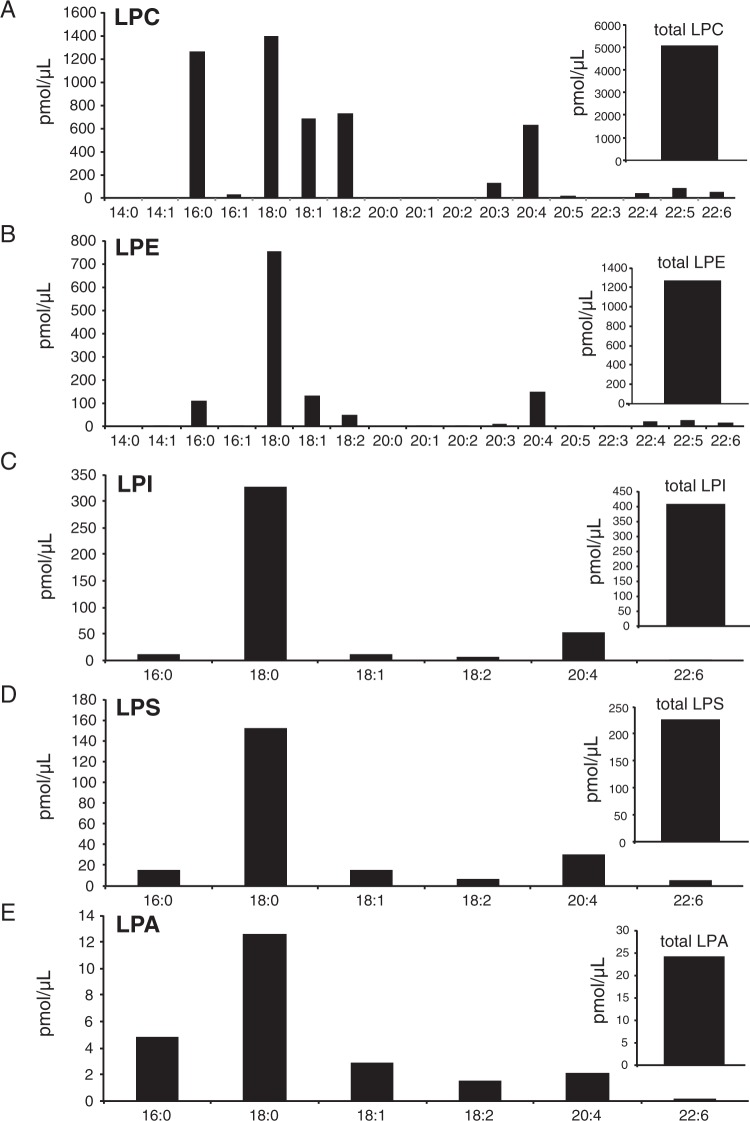
Liquid chromatography electrospray ionization tandem mass spectrometric (LC-MS/MS) analysis of porcine liver decomposition product (PLDP) lyso-phosholipid (LPL) composition. LPL species in PLDP were identified (via selected reaction monitoring), including (**A**) lysophosphatidylcholine (LPC), (**B**) lysophosphatidylethanolamine (LPE), (**C**) lysophosphatidylinositol (LPI), (**D**) lyophosphatidylserine (LPS), and (**E**) lysophosphatidic acid (LPA).

An analysis of the composition of PLDP revealed that the most abundant PLs and LPLs belonged to the PC class. Within this class, PC was the most abundant PL (7,100 pmol/μL), followed by PE (2,200 pmol/μL). Other identified PLs included PS (120 pmol/μL), PI (200 pmol/μL), and PA (3.9 pmol/μL). As shown in Fig. 3, the PC species detected (via ion chromatogram extraction (EIC)) at high abundance in the PLDP included PC34:1, PC34:2, PC36:1, PC36:2, PC36:3, PC36:4, and PC38:4, while others (such as PC32:0, PC32:1, PC38:3, PC38:5, PC38:6, PC40:4, PC40:5, and PC40:6) were also detected at lower concentrations. The PE species detected at the highest abundance included PE36:2 and PE38:4, (those detected at lower concentrations included PE34:1, PE34:2, PE36:1, PE36:3, PE36:4, PE38:3, PE38:5, PE38:6, PE40:4, PE40:5, and PE40:6), while PI38:4 was measured to occur most abundantly of the PI species, (PI34:1, PI34:2, PI36:1, PI36:2, PI36:3, PI36:4, PI38:3, PI38:5, PI40:4, and PI40:5 were also detected at lower concentrations). The most abundant of the PS species included PS36:1 and PS38:4, (PS34:1, PS34:2, PS36:2, PS36:3, PS36:4, PS38:3, PS38:5, PS38:6, PS40:4, PS40:5, and PS40:6 were detected at lower concentrations), while the most abundant PA species were PA36:1 and PA38:4, (PA32:0, PA32:1, PA34:0, PA34:1, PA34:2, PA36:0, PA36:2, PA36:3, PA36:4, PA38:3, PA38:5, PA38:6, PA40:4, PA40:5, and PA40:6 were detected at lower concentrations). Likewise, the conducted EIC analysis of LPCs in the PLDP showed that the most abundant LPC (total, 5,000 pmol/μL) was LPC18:0, (LPC16:0, LPC18:1, LPC18:2, and LPC20:4 were also detected) (Fig. 4). The most abundant LPE (total, 1,200 pmol/μL) was LPE18:0 (LPE16:0, LPE18:1, LPE18:2, and LPE20:4 were also detected), while the most abundant LPI (total, 400 pmol/μL) detected was LPI18:0 (LPI16:0, LPI18:1, LPI 18:2, and LPI20:4 were also detected). The most abundant LPS (total, 230 pmol/μL) detected was LPS18:0, (LPS16:0, LPS18:1, LPS18:2, LPS20:4, and LPS22:6 were detected at lower concentrations), and the most abundant LPA (total, 25 pmol/μL) detected was LPA18:0, (while LPA16:0, LPA18:1, LPA18:2, and LPA20:4 were detected at lower levels).

### PLDP-derived LPLs concentration-dependently modulate microglial activation *in vitro*

**Figure 5:**
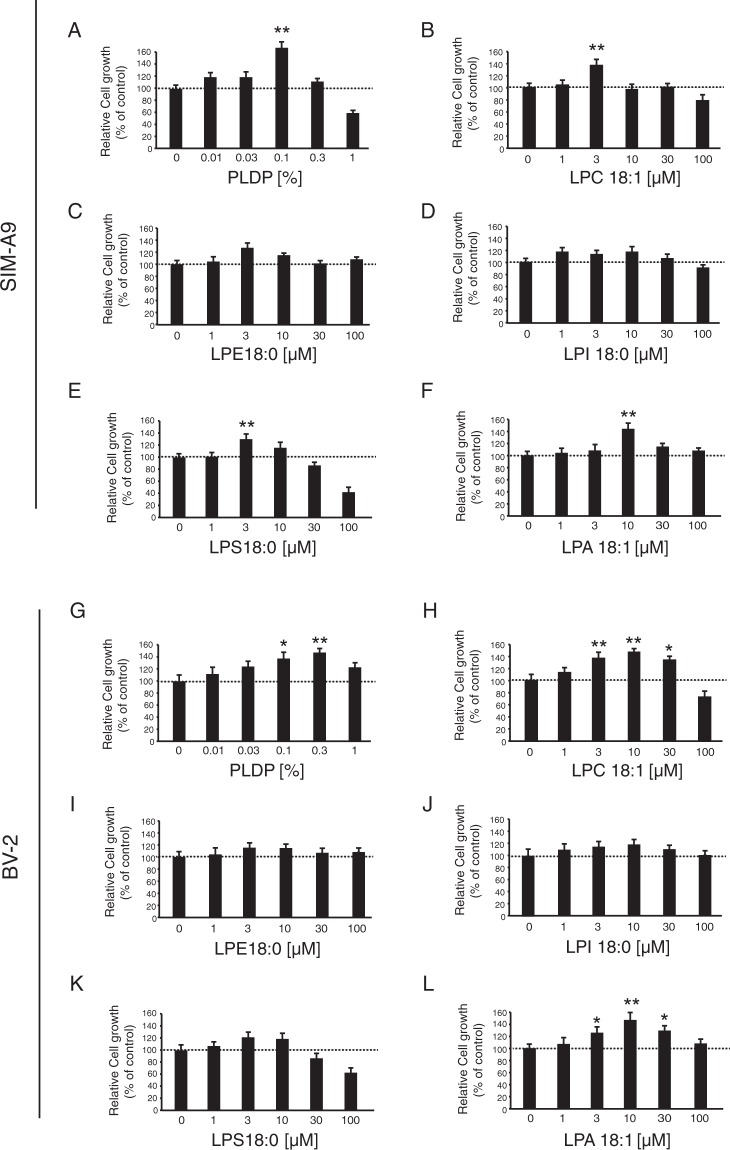
Effect of porcine liver decomposition product (PLDP)-derived lyso-phosholipid (LPLs) on SIM-A9 and BV-2 cell proliferation. Microglial cells were assess using an MTT colorimetric assay after treatment with (**A,G**) PLDP, (**B,H**) lysophosphatidylcholine (LPC)18:1, (**C,I**) lysophosphatidylethanolamine (LPE)18:0, (**D,J**) lysophosphatidylinositol (LPI)18:0, (**E,K**) lyophosphatidylserine (LPS)18:0, or (**F,L**) lysophosphatidic acid (LPA)18:1 for 24 h. Data are expressed as the mean ± S.E. n = 3, **P < 0.01 or *P < 0.05.

Next, we characterized microglia activation (including cell shape, proliferation, cell morphology, and gene expression profiles) as induced via the administration (24 h) of PLDP or individual LPLs (Fig. 5A–F). Treatment of SIM-A9 cells (that were cultured in serum-free medium) with moderate concentrations (0.01–0.03%) of PLDP caused a marked increase in cell proliferation, whereas exposure to high concentrations (1%) of PLDP inhibited cell proliferation. Similarly, a conducted MTT spectrophotometric assay revealed that moderate concentrations of all five LPLs stimulated SIM-A9 cell proliferation; however, high concentrations (100 μM) of LPS (lysophosphatidylserine) or LPC significantly decreased proliferation, while high concentrations of LPE, LPI, and LPA did not have any statistically significant effect on cell proliferation compared to controls. Similarly, PLDP treatment significantly increased BV2 cell proliferation in a concentration-dependent manner; however, while treatment with low levels of LPC or LPA (<30 μM) also significantly increased BV2 proliferation, treatment with concentrations of LPS (lysophosphatidylserine) or LPC ≥30 μM had a cytotoxic effect (Fig. 5G–L).

### LPC stimulates autotaxin (ATX) secretion to modulate microglial activation

**Figure 6:**
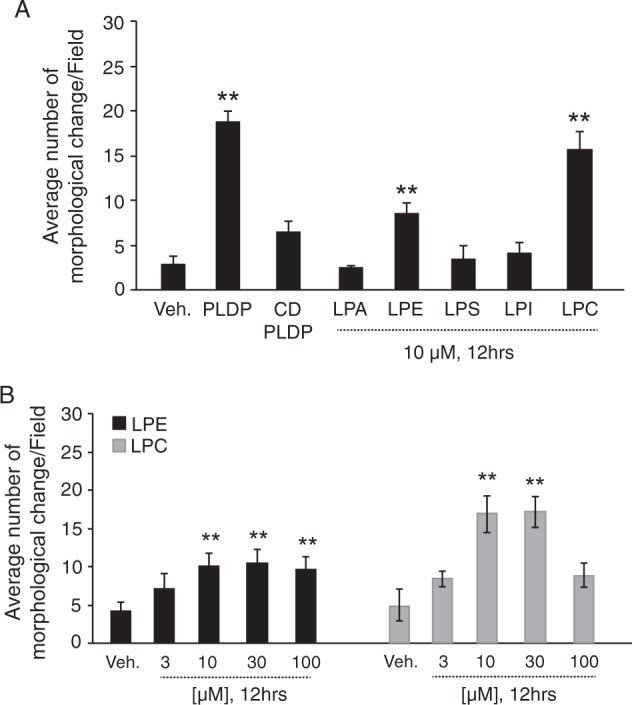
Induction of morphological change by lysophosphatidylcholine (LPC) or lysophosphatidylethanolamine (LPE) in SIM-A9 cells. (**A**) Microglial cells were exposed to serum-free medium containing vehicle, PLDP (1%), charcoal-dextran-treated 1% PLDP (CD-PLDP) or 10 μM each LPLs (LPA, LPE, LPS, LPI, and LPC for 12 h. The number of cells exhibiting morphological change was measured. Representative data from three independent experiments are shown. Magnification, ×200. (**B**) The number of cells exhibiting morphological change was measured after vehicle or 30 µM LPC or LPE for 12 h. Cells were exposed to serum-free medium containing vehicle or the indicated concentration of LPC or LPE for 12 h. Representative data from three independent experiments are shown. Magnification, ×200. Data are expressed as the mean ± S.E. *P < 0.01).

**Figure 7:**
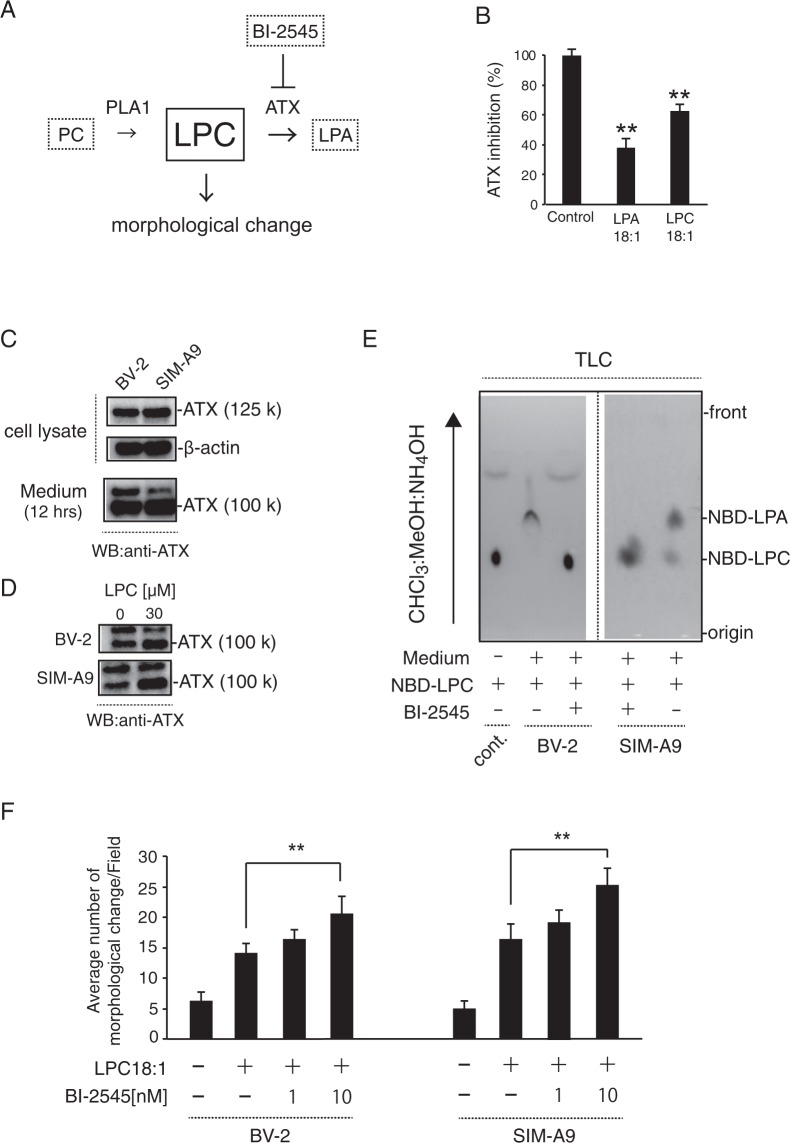
The autotaxin (ATX) inhibitor BI-2545 promotes lysophosphatidylcholine (LPC) -mediated cell morphology. (**A**) Enzymatic pathways controlling lysophosphatidic acid (LPA) and LPC levels. ATX cleaves LPC to generate LPA, and then PLA_1_ catalyzes the hydrolysis of PC to LPC. (**B**) LPC (18:1) inhibited ATX activity. Both LPC and LPA (each 10 μM) inhibition of ATX were significantly decreased. Data are expressed as the mean ± S.E. *P < 0.01) (**C**) ATX levels in a concentrated (∼30-fold) microglial-conditioned culture medium (upper panel) and cell lysate (lower panel) were analyzed via western blotting using a rabbit anti-ATX polyclonal antibody. (**D**) BV-2 and SIM-A9 both cell line was treated with or without LPC (30 μM) for 12 h. LPC-mediated ATX expression was analyzed via western blotting using a rabbit anti-ATX polyclonal antibody. (**E**) Thin-layer chromatography (TLC) analysis of NBD-LPA levels after treatment with concentrated conditioned medium, with or without BI-2545 (10 nM) for 12 h. Samples aliquots (10 μl) were spotted onto the TLC plate. NBD-LPC 18:1 or NBD-LPA 18:1 were detected using blue LED irradiation. The microglial-conditioned medium generated NBD-LPA18:1 at expected levels, but this effect was completely abolished by the addition of BI-2545. (**F**) BI-2545 treatment promoted LPC-mediated (10 μM) cell morphology. Neurite lengths were measured for 100 cells, and the total neurite length/cell was calculated. Data are presented as the mean ± S.E. *P < 0.01.

We next examined the mechanisms underlying the effect of the various LPLs on cell morphology. As shown in Fig. 6A,B, PLDP-, LPC- and LPE-treated cells exhibited markedly faster cell morphology induction than those that were treated with LPA, LPS, or LPI, and also exhibited morphological change and network formation after 12 h of treatment. (In contrast, the majority of LPA-treated cells maintained round shapes). We showed that maintaining PLDP in the presence of charcoal-dextran-treated caused reduced morphological change of SIM-A9 cells. Notably, the induction of morphological change in the LPE- and LPC-treated cells occurred in a concentration-dependent manner, and LPC, but not LPE, significantly induced morphological change. The ecto-enzyme ATX cleaves LPC to generate LPA^39^ (Fig. 7A); thus, we next assessed its potential role in, (i.e. whether ATX-mediated LPC degradation caused) the observed reduced morphological change. As shown in Fig. 7B, *in vitro* ATX assay was performed. We found that ATX lysoPLD is inhibited by 10 μM LPC (18:1) which decreased up to 60%. We also examined expression levels in the microglial cells via a western blot analysis, and resultantly detected ATX in both the collected cytosolic and the extracellular fractions (Fig. 7C). Moreover, LPC exposure to increased ATX levels in medium was shown in Fig. 7D. This finding supports that LPC administration likely stimulates ATX secretion.

### ATX inhibition promotes morphological change

Since a previous study reported that treatment with the NBD-labeled LPC18:1, ATX inhibitor BI-2545 (Boehringer Ingelheim) significantly reduced LPA levels *in vivo*^40^, we next assessed its effect on LPA levels in the present study. As shown in Fig. 7E, while microglial-conditioned medium contained expected levels of LPA, these were completely abolished by the addition of 100 nM BI-2545. Moreover, BI-2545-treated cells were induced to exhibit markedly faster morphological change than LPC18:1-treated cells after 12 h (Fig. 7F). Together, these and the previously reported findings suggest that inhibiting ATX production promotes morphological change.

### Effect of PLDP and LPC on LPS (lipopolysaccharide)-induced cytokines mRNA in microglial cells

**Figure 8:**
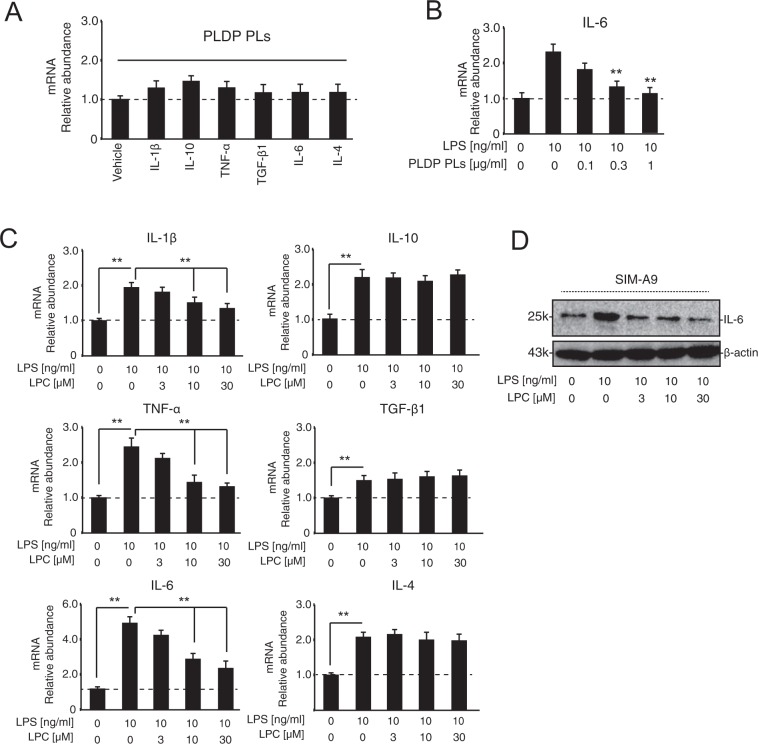
Pro- and anti-inflammatory cytokine mRNA expression by SIM-A9 cells after exposure to lipopolysaccharide (LPS), with or without lysophosphatidylcholine (LPC). (**A**) SIM-A9 cells were treated with PLDP-derived PLs (0.3 μg/ml) for 24 h, before their relative mRNA expression of interleukin *(IL)-1β*, *tumor necrosis factor (TNF)-α, IL-4, IL-6, IL-10, IL-12*, and *transforming growth factor (TGF)-β* was assessed. Data are expressed as the mean ± S.E. n = 3, **P < 0.01. (**B**) SIM-A9 cells were treated with LPS (10 ng/ml) with or without PLDP-derived PLs (0.3 μg/ml) for 24 h, before their relative mRNA expression of *IL-6* was assessed. Data are expressed as the mean ± S.E. n = 3, **P < 0.01. (**C**) SIM-A9 cells were treated with LPS (10 ng/ml) with or without 0, 1, 3, 10, or 30 μM LPC for 24 h, before their relative mRNA expression of interleukin *(IL)-1β*, *tumor necrosis factor (TNF)-α, IL-4, IL-6, IL-10, IL-12*, and *transforming growth factor (TGF)-β* was assessed. Data are expressed as the mean ± S.E. n = 3, **P < 0.01. (**D**) SIM-A9 cells were treated with LPS (10 ng/ml) with or without 0, 3, 10, or 30 μM LPC for 24 h. LPS-mediated IL-6 expression was analyzed via western blotting using a rabbit anti-IL-6 polyclonal antibody.

We next explored the effect of PLDP and LPC on LPS-induced pro-inflammatory cytokine expression. As shown in Fig. 8A, PLDP-derived PLs was not significantly different of expression of pro-inflammatory and anti-inflammatory cytokines. We also measured LPS-mediated IL-6 expression with or without PLDP-derived PLs treatment, IL-6 expression was dose dependently decreased (Fig. 8B). As shown in Fig. 8C, SIM-A9 cells were simultaneously exposed to LPS (10 ng/ml), with or without LPC (3, 10, and 30 μM) at 24 h, before cytokine mRNA expression was assessed via a real-time PCR analysis. The addition of LPC attenuated the increased expression of the pro-inflammatory cytokines, interleukin (IL)-1β, IL-6, and tumor necrosis factor (TNF)-α. For the anti-inflammatory cytokines including IL-10, TGF-β, and IL-4, the effect of LPC did not change upon the LPS exposure. As shown in Fig. 8D, we measured LPS-mediated IL-6 expression with or without LPC treatment, IL-6 expression was dose dependently decreased. This finding supports that PLDP-derived LPLs have same activity.

## Discussion

Many previous reports have suggested that dietary PLs such as PC, PS, and SM are critical to maintain brain development and cognitive performance^12,41,42^. These lipids contain two fatty acids that are ester-linked to glycerol at sn-1 and sn-2, and a polar head group that is held at sn-3 by a phosphodiesterase bond^43^. The major classes of glycerophospholipids comprise PA, phosphatidylglycerol (PG), PI, CL, and aminoglycerophospholipids, including PC, PE, and PS^44^. In present study, we focused on newly identified bioactive PLs, such as LPLs, which are produced by activated platelets, damaged cells, and cells that have been stimulated with growth factors. Over the past few decades, various molecular pathways that regulate microglial activation, as well as the effects of activated microglia on neurons, have been identified^45^. Activated microglial cells release diverse substances such as reactive oxygen species, cytokines, and growth factors that contribute to dementia pathogenesis in both the acute and chronic phase, as well as during subsequent regeneration^46–49^. Data from the current LC-MS/MS study showed that PLDP contains a complex range of LPLs; however, their effects on cognitive function or mental performance have not been previously confirmed. To explore whether and how PLDP and LPLs stimulate microglial activation, we tested their effects on microglial proliferation (which is a key component of the brain’s damage response), cell morphology, and cytokine expression *in vitro*. We previously showed that PLDP-derived LPLs promote microglial activation and induce morphological change *in vitro*. Importantly, other research groups have suggested the proliferative and neurite-outgrowth properties exhibited by microglia represent a promising potential therapeutic target to repair injuries or treat degenerative disorders of affecting the central nervous system (CNS)^50^. This is consistent with their role during development, when dynamic patterns of morphological change during facilitate the formation of complex CNS and brain neuronal networks^51^, as well as their established roles in synapse monitoring^52^, apoptotic-debris clearing, and synaptic pruning^53^, all of which are required to maintain normal brain development and CNS homeostasis^54^. Microglia rapidly alter their morphology and function in response to changes in the brain microenvironment^55^. In the present study, we showed that media conditioned via exposure to LPC- or LPE-treated microglia promoted morphological change in a dose-dependent manner. LPC is an intermediate in multiple lipid metabolic pathways, and circulates in the bloodstream at a much higher concentration than LPA^56^, reaching approximately 190 μM in human plasma, and up to 800 μM in the blood plasma of other mammalian species^57^. In contrast, the level of LPC in the cerebrospinal fluid is only approximately 5 μM, since ATX is abundantly produced in cerebrospinal fluid, and converts LPC to LPA^58,59^. Previous reports have suggested that LPA mediates a diverse array of biological responses including mitogenesis, cell morphology, and cytoskeletal organization, including the retraction of neurites from neuronal cells^60–62^. It activates multiple molecular targets, including six G protein-coupled receptors, which in turn stimulate various signaling pathways^63,64^. In the present study, we identified that LPC, but not LPA, promoted morphological change *in vitro*, consistent with a previous report which demonstrated that LPC induces morphological change in PC12 cells by activating phospholipase D_2_^65^. Interestingly, the ATX inhibitor BI-2545 also significantly promoted morphological change in BV-2 and SIM-A9 cells, which suggests that LPC may promote morphological change by inhibiting ATX. Macrophage and microglial activation has been shown to lead to two opposing cell states, namely ‘classic (M1)’ and ‘alternative (M2)’^66^. The M1 phenotype is considered to be a pro-inflammatory state, in which microglial cells produce and release ROS and cytokines. Conversely, the M2 microglial phenotype is considered to be an anti-inflammatory state associated with the production and release of trophic factors, such as transforming growth factor (TGF)-β, and brain-derived neurotrophic factor (BDNF)^66^. In the present study, LPC treatment attenuated the observed increased expression of the pro-inflammatory cytokines IL-1β, IL-6, and TNF-α, which suggests that it may be neuroprotective, and/or protect against neuroinflammation. Indeed, previous studies have suggested that the pro-inflammatory cytokine TNF-α is a primary mediator of the inflammatory response that stimulates the synthesis and release of other cytokines^67^. Furthermore, previous study also suggested that sensitivity to the anti-inflammatory and M2 promoting cytokines interleukin (IL)-10 and IL-4^68^. In BV-2 microglia cells, IL-4, but not IL-10, re-directed LPS (lipopolysaccharide)-activated microglia towards an M2 phenotype^68^. These cytokines which are influenced by regulatory T cells and prevent inflammatory processes^69^. Our results showed that the LPS-mediated inflammatory response, including the observed increase in microglial cytokine production, was suppressed by LPC exposure. This finding is important given that microglia-mediated neuroinflammation is regarded as a pathological mechanism in many neurodegenerative diseases, and a key event accelerating cognitive or functional decline.

## Conclusions

Consistent with our previous findings, the present study demonstrated that PLDP and PLDP-derived LPLs concentration-dependently modulate microglial activation *in vitro*. Furthermore, we herein showed that LPC concentration-dependently promotes cell morphology, likely via effects mediated by ATX, since inhibiting ATX also promotes cell morphology while conversely, increasing ATX production (via treatment with LPC) abolishes this effect. These findings support that LPC is likely neuroprotective, and thus, is a promising therapeutic candidate to treat age-related cognitive impairments such as dementia. Further studies are warranted to investigate LPC in the regulation of cognition, and the reversal of age-associated cognitive decline *in vivo*.

## Supplementary information

Supplementary Information.
