# Supplementary material for: Porcine liver decomposition product-derived lysophospholipids promote microglial activation in vitro
Source: Sci Rep. 2020 Feb 28;10:3748. doi: 10.1038/s41598-020-60781-1 (PMC7048828; doi:10.1038/s41598-020-60781-1)
Supplement: Supplementary file 1 — Supplementary Information. [file 41598_2020_60781_MOESM1_ESM.pdf]

Porcine liver decomposition product-derived lysophospholipids promote microglial activation *in vitro*

Supplementary Material

Tamotsu Tsukahara[a*], Hisao Haniu[b], Takeshi Uemura[b,c], and Yoshikazu Matsuda[d]

[a]Department of Pharmacology and Therapeutic Innovation, Nagasaki University Graduate School of Biomedical Sciences, 1-14 Bunkyo-machi, Nagasaki 852-8521, Japan

[b]Institute for Biomedical Sciences, Shinshu University Interdisciplinary Cluster for Cutting Edge Research 3-1-1 Asahi, Matsumoto, Nagano 390-8621, Japan

[c]Division of Gene Research, Research Center for Supports to Advanced Science, Shinshu University 3-1-1 Asahi, Matsumoto, Nagano 390-8621, Japan

[d]Division of Clinical Pharmacology and Pharmaceutics, Nihon Pharmaceutical University, Ina-machi, Saitama 362-0806, Japan

Supplemental data (Gel image)

Cytosolic fraction

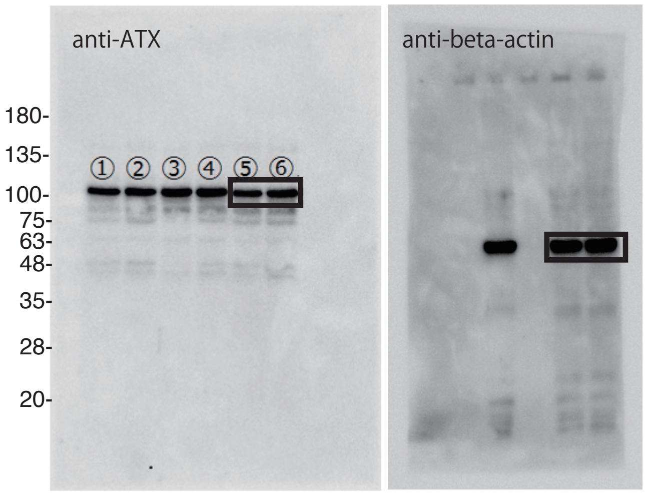

10% SDS-PAGE

S. Fig.7C (Full-length blot, Fig7C, upper)

Culture medium

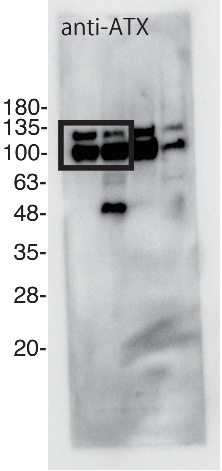

10% SDS-PAGE

S. Fig.7C (Full-length blot, Fig7C, lower)

Culture medium

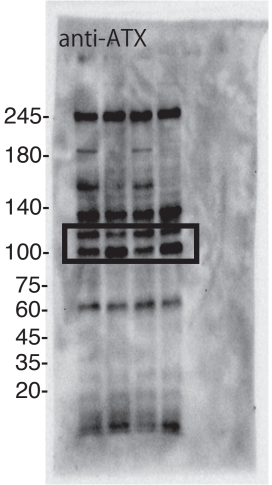

4-20% SDS-PAGE

S. Fig.7D (Full-length blot, Fig7D)

SIM-A9 cell

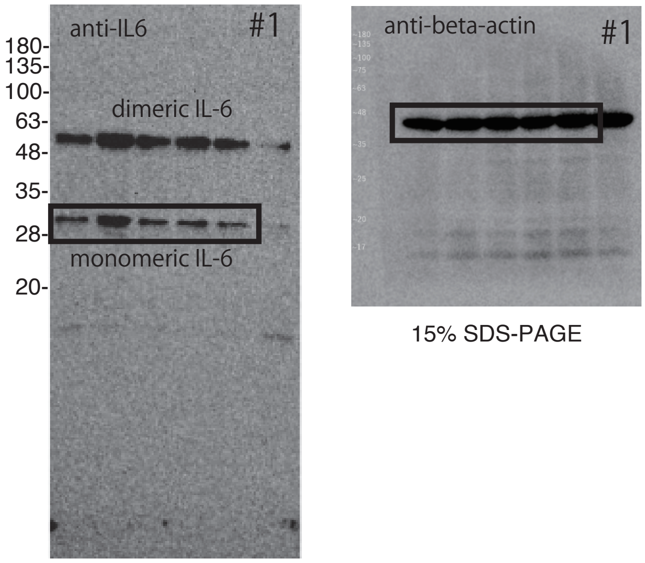

15% SDS-PAGE

15% SDS-PAGE

S. Fig.8D (Full-length blot, Fig8D)

BV-2 cell

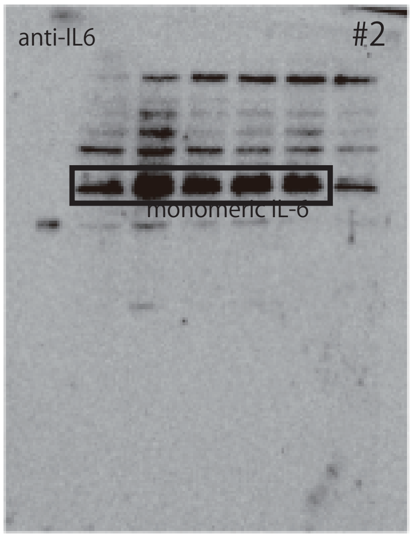

15% SDS-PAGE

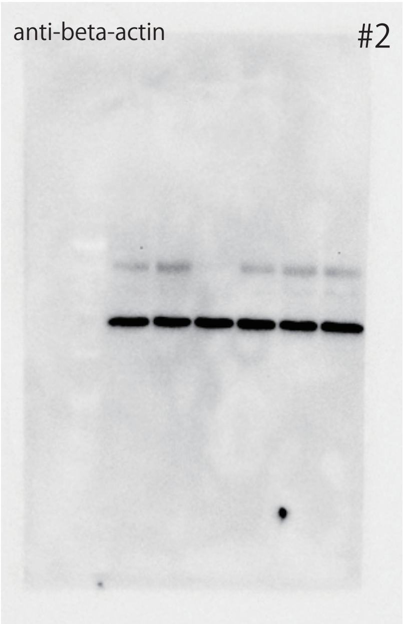

10% SDS-PAGE
